# The Potential Role of Cellular Senescence in Non-Alcoholic Fatty Liver Disease

**DOI:** 10.3390/ijms23020652

**Published:** 2022-01-07

**Authors:** Cornelius Engelmann, Frank Tacke

**Affiliations:** 1 Department of Hepatology and Gastroenterology, Campus Virchow-Klinikum (CVK) and Campus Charité Mitte (CCM), Charité—Universitätsmedizin Berlin, 13353 Berlin, Germany; frank.tacke@charite.de; 2 Berlin Institute of Health (BIH), 10178 Berlin, Germany; 3 Institute for Liver and Digestive Health, University College London, London NW3 2PF, UK

**Keywords:** NAFLD, NASH, SASP, senescence associated secretory phenotype, mitochondrial dysfunction, fibrosis

## Abstract

Non-alcoholic fatty liver disease (NAFLD) represents an increasing global health burden. Cellular senescence develops in response to cellular injury, leading not only to cell cycle arrest but also to alterations of the cellular phenotype and metabolic functions. In this review, we critically discuss the currently existing evidence for the involvement of cellular senescence in NAFLD in order to identify areas requiring further exploration. Hepatocyte senescence can be a central pathomechanism as it may foster intracellular fat accumulation, fibrosis and inflammation, also due to secretion of senescence-associated inflammatory mediators. However, in some non-parenchymal liver cell types, such as hepatic stellate cells, senescence may be beneficial by reducing the extracellular matrix deposition and thereby reducing fibrosis. Deciphering the detailed interaction between NAFLD and cellular senescence will be essential to discover novel therapeutic targets halting disease progression.

## 1. Introduction

Non-alcoholic fatty liver disease (NAFLD) is one of the major global non-communicable diseases affecting 25–30% of the population in Western countries [1]. It is one of the leading indications for liver transplantation and has shown a 7-fold increase between 2002 and 2016 according to the European Liver Transplant Registry (ETLR) [2]. The term NAFLD spans a spectrum of disease conditions ranging from non-alcoholic fatty liver (NAFL) to non-alcoholic steatohepatitis (NASH) and NASH-related cirrhosis [1]. The transition from NAFL to NASH identifies a more aggressive disease phenotype with increased likelihood of developing complications such as cirrhosis and also hepatocellular carcinoma (HCC) [1].

It is believed that 7–30% of those with NAFLD fulfill criteria for NASH [3,4]. The risk of developing HCC from NASH is estimated to be 5.29 per 1000 people (95%CI: 0.75–37.56) [5] and 10.6 per 1000 people years from NASH cirrhosis [6]. It is apparent that NAFLD is associated with metabolic syndrome and type 2 diabetes especially when NAFLD is progressing. However, some studies reported that NAFLD develops in 10–20% of non-obese people, so-called lean NAFLD [4,7]. Recently, a new term called metabolic-dysfunction-associated fatty liver disease (MAFLD) was introduced to describe more appropriately the spectrum of pathomechanistic changes and to prevent the simple dichotomous classification to steatohepatitis and non-steatohepatitis [8].

**Figure 1:**
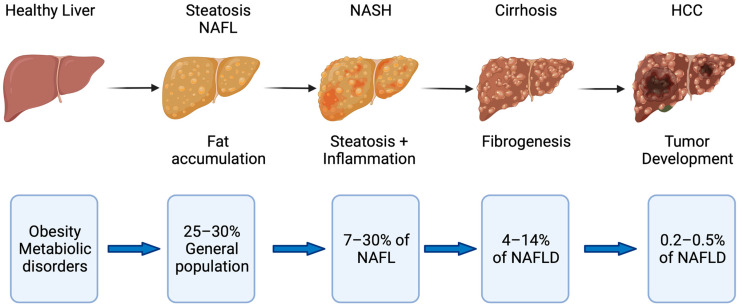
Disease progression in fatty liver disease. Obesity and metabolic disorders represent risk factors for NAFLD, which starts with fat accumulation in hepatocytes. Further disease progression is characterized by inflammation (NASH) and subsequent fibrogenesis leading to cirrhosis. NASH with and without cirrhosis bears the risk of developing hepatocellular carcinoma (HCC) [5,6,20].

Although cardiovascular events and malignancies represent the most frequent causes of death within the NAFLD cohort, liver-related complications and deaths are still far higher than in the normal population (Figure 1) [9]. A recently published trial in 1339 biopsy-proven NAFLD patients indicated that non-obese or lean patients with NAFLD have a similar liver-related and overall mortality compared with obese NAFLD patients [10]. Targeting NAFLD with the aim to prevent disease progression may be an incremental component of patient management strategies. However, most of the recent phase 3 trials testing novel compounds have been prematurely terminated and as of now there is still no specific treatment approved for NAFLD [11].

One starting point of NAFLD is that hepatocytes are not capable of metabolizing all energy substrates such as carbohydrates and fatty acids leading to accumulation of toxic lipids [12]. Hepatocyte fat accumulation and inflammation are the main drivers of disease progression [12,13] and the subsequent hepatocellular injury is characterized by alterations of the cellular function. Endoplasmic reticulum stress [14] and activation of unfolded protein response [15] stimulate apoptotic pathways leading to cell death [16]. Inflammatory pathways in hepatocytes (such as Nf-kB or inflammasome activation) and release of DAMPs (damage-associated molecular patterns) results in stimulation of neighboring non-parenchymal cells and signaling via pattern recognition receptors (PPR) such as toll-like receptors (TLR). Subsequent secretion of cytokines and chemoattractants (e.g., CCL2) trigger recruitment of immune cells such as monocytes/macrophages and activate stellate cells leading to fibrosis [17,18,19]. These pro-inflammatory and pro-fibrotic processes mark the transition from NAFL to NASH. It is of major interest to identify pathomechanistic elements involved in the broader disease spectrum and progression potentially serving as ideal therapeutic targets for patients with NAFLD at different disease stages.

Cellular senescence is a term describing cells which are in cell cycle arrest but remain metabolically active [21]. There is evidence showing that cellular senescence, especially in hepatocytes, may modulate fat accumulation and inflammation in patients with different stages of NAFLD [21,22]. Therefore, this review will focus on the current evidence for the role of cellular senescence in NAFLD and its effect on intercellular crosstalk as a central driver of disease progression.

## 2. The Role of Cellular Senescence in Fatty Liver Disease

### 2.1. Mechanisms of Cellular Senescence in the Liver 

Cellular senescence is a term which generally marks a decline of cell division capacity and ability to proliferate [21]. In the context of mutations, senescence is considered protective since it prevents the unlimited proliferation and dissemination of potentially malignant cells [23]. Cellular senescence may be also linked to hepatic injury. In some cell types such as fibroblast or hepatic stellate cells, senescence may be beneficial as it mitigates scar formation and fibrosis [21]. In addition, senescence is an essential process during maturation that ensures coordinated and controlled growth [24]. 

Initially, cellular senescence was recognized as the consequence of telomere shortening (telomere-dependent senescence) after multiple cycles of cell division and during aging. Cellular senescence also develops as the consequence of DNA damage, oncogene expression, reactive oxygen species and other types of cellular injury that cause a telomere-independent form of senescence [22]. Mitochondrial dysfunction may also lead to cellular senescence, and mitochondrial-dysfunction-associated senescence (MiDAS) is closely linked to alterations of cell metabolisms [25].

Although senescent cells are no longer able to proliferate, they remain metabolically active and may communicate with surrounding cells, thereby spreading senescence, inducing injury and initiating immune cell recruitment through the senescence-associated secretory phenotype (SASP) [26,27,28,29]. Senescent cells contribute to chronic inflammation, may promote tumor growth by stimulating angiogenesis through SASP and prevent regeneration after injury [21]. 

**Figure 2:**
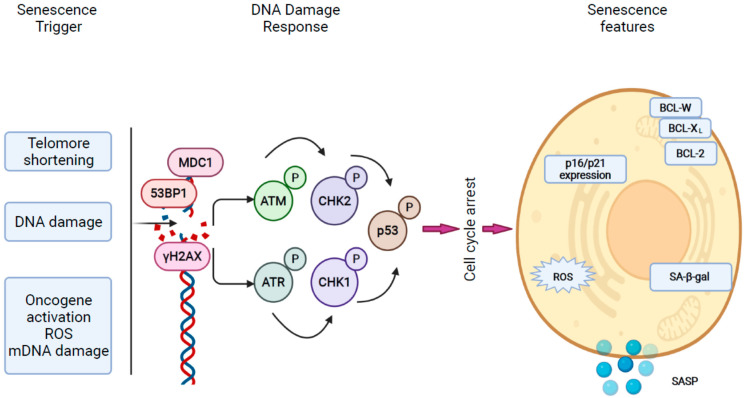
Mechanisms of cellular senescence. Cellular senescence can have various triggers such as telomere shortening, DNA damage, oncogene activation and mitochondria-related factors such as reactive oxygen species (ROS) and mitochondrial DNA damage. Upon injury, the DNA damage response (DDR) mechanisms are activated and signal transduction through protein phosphorylation of ATM, ATR, CHKs stabilizes p53, thus inducing cell cycle arrest through RB. Senescence cells show high expression of p16 and p21 and high activity of SA-β-gal and secrete cytokines and chemoattractants via SASP (modified from Di Micco R et al. [30]).

Senescence develops as the result of tissue injury, which activates the DNA damage response (DDR) pathway. DNA damage may occur as the consequence of direct double-strand breaks within the telomere region and also after oncogenic activation, leading to hyperproliferation, replication stress and DNA damage at fragile sites [30]. Upon injury, the DDR forms a complex of DNA response factors, including phosphorylated H2AX (γH2AX), MDC1, 53BP1 and activated ataxia telangiectasia mutated (ATM), creating foci surrounding the damaged areas [30]. ATM activates and stabilizes downstream tumor suppressor oncogenes, which induce a cell cycle arrest. In humans there are two types of cyclin-dependent kinase (CDK) inhibitors: INK4, which involves p16^INK4A^ (CDKN2A) and the Cip/Kip family, majorly controlled by p53 and further downstream by p21^WAF1/Cip1^ (CDKN1A). These factors induce cell cycle arrest by maintaining the hypophosphorylated form of retinoblastoma family members (RB) [21]. Some observations report an overexpression of anti-apoptotic BCL family proteins (BCL-XL, BCL-2, BCL-W) [31,32] and metabolic changes also include activation of β-galactosidase (senescence-associated β-galactosidase (SA-β-gal) [33] (Figure 2)). 

SASP is a crucial aspect of senescence, which exerts significant effects on surrounding cells such as immune cell recruitment, spread of senescence and modulation of extracellular matrix. The SASP program includes multiple secretory factors of which the pro-inflammatory cytokines interleukin-6 (IL-6), CXC chemokine ligand 8 (CXCL8 or IL-8), monocyte chemoattractant protein 1 (MCP1 or CCL2) and other proteins such as angiopoietin-like 2 (anglptl2) are the most important [30,34]. Circulating anglptl2 levels were observed to be increased in various age-related and metabolic diseases, proposing anglptl2 as a potential biomarker for senescence-related processes [34]. Matrix metalloproteinases (MMPs), serine/cysteine proteinase inhibitors (SERPINs) and tissue inhibitors of metalloproteinases may be involved in remodeling of extracellular matrix, and exosomes may induce senescence in adjacent cells and may be pro-tumorigenic [30].

SASP activation is slow and requires persistent DDR signaling, but factors involved in mediating cell cycle arrest such as p16, p53 and p21 are not involved. Upstreaming DDR elements ATM, NBS1 and CHK2 promote the paracrine function of senescent cells [35]. SASP activity is majorly controlled at transcriptional level through NF-kB and CCAAT/enhancer-binding protein-b (C/EBPb). GATA4 activates the transcription of effector molecules in an IL-1a-dependent manner [36]. 

### 2.2. Cellular Senescence in Humans with NAFLD

Several observations in humans strongly indicate the (potential) involvement of cellular senescence in NAFLD. Generally, livers from humans with fatty liver disease overexpress p53 and have diminished BCL-2 expression, a global indicator for cellular senescence and apoptosis [37]. 

In a study including 70 patients with type 2 diabetes mellitus and no NAFLD at baseline, 39 patients developed NAFLD over a period of 6 years. These patients had shorter telomeres after 6 years despite having the same age and telomere length at baseline [38]. Another study analyzed telomere length and activity of the telomerase reverse transcriptase in peripheral lymphocytes in an age-matched cohort of patients with NAFLD (n = 22), cryptogenic cirrhosis (n = 20) and healthy individuals (n = 20). Shorter telomere length and a high number of cells with hyper fragmented chromatin, so-called senescence-associated heterochromatin foci (SAHF), were observed in NAFLD, significantly more often in comparison to cryptogenic cirrhosis. Expression of telomerase reverse transcriptase (TERT), which adds nucleotides to the chromosome’s telomeres to stabilize senescent cells [39], was significantly higher in NAFLD [40]. 

NAFLD-related telomere shorting in hepatocytes was observed to be associated with DNA damage leading to an increased yH2AX expression and high number of cells in cell cycle arrest indicated by high p21 expression. Higher yH2AX expression was associated with more severe liver steatosis and hepatocyte p21 expression correlated with the degree of liver fibrosis and development of diabetes mellitus. Cellular senescence was also associated with adverse liver-related outcomes such as hepatocellular carcinoma, liver transplantation or liver-related deaths [41]. p53-binding protein 1 (53BP1) is, besides yH2AX, another DNA damage response (DDR) protein that binds quickly to sites of DNA damage, especially double strand brakes. In human liver tissue from NAFLD patients, 53BP1-foci formation in hepatocytes was significantly higher than in controls. These foci were found more often in patients with NASH than in NAFL and correlated with the degree of fibrosis [42]. DNA damage in association with NASH does not seem to be restricted to genomic DNA but may also occur in mitochondria. In 252 liver specimens from NAFLD patients mitochondrial dysfunction and mitochondrial DNA damage were associated with more severe forms of NAFLD and with inflammation [43]. Therefore, several authors proclaimed genomic instability to play an essential role during disease progression in NAFLD. 

DNA methylation represents a form of epigenetic regulation, which is also considered as a marker for aging and cellular senescence. Several studies have identified a multitude of methylated genes, including genes encoding for metabolism (e.g., PC, ACLY, PLCG1) and for insulin signaling (e.g., IGF1, IGFBP2, PRKCE), as indicators for disease initiation or progression [44]. These alterations in gene methylation profiles were partially reversible after bariatric surgery [44]. DNA methylation primarily affects diverse methylation networks, which become apparent during NAFLD progression and also during aging. The first network involves downregulated genes for transcriptional regulation and regeneration/proliferation, and the second network contains regulated genes for lipid metabolism [45]. Another study identified DNA methylation patterns related to genes involved in fibrogenesis, especially in those patients with rapidly progressing disease and high grades of fibrosis [46]. As the consequence of all these positive results, plasma DNA methylation was proposed as a potential biomarker for NASH-related fibrosis [47]. 

Single-nucleotide polymorphisms (SNP) of signaling molecules in senescence pathways may alter their signaling activity and may contribute to the development of cellular senescence. CDKN1A is the gene encoding for p21, and CDKN1A SNPs were shown to be potentially related to disease progression in NAFLD. Variant rs762623, where substitution of the G allele with the A allele leads to lower expression of p21, correlated with the occurrence of cirrhosis. This fact, which is against the known paradigm of cellular senescence as a driver of NAFLD, highlights the complex mechanisms and effects cellular senescence may have in advanced disease stages, where multiple cell types, also including stellate cells as the major ECM producers, are involved [48] (Table 1). 

### 2.3. Mechanisms of Disease Progression in NAFLD

#### 2.3.1. The Role of Cellular Senescence in Hepatocyte Fat Accumulation 

The first phase of NAFLD is related to fat accumulation in hepatocytes. NAFLD prevalence increases in older age, suggesting that there might be a link between fat accumulation and cellular senescence [49]. In rat models of fatty liver disease, animals with more enhanced subcutaneous fat tissue and liver fat gain, categorized as obesity-prone, showed higher mRNA levels of p16 and p21 but lower expression of p53 and phosphorylated retinoblastoma 1 (Rb1) [50]. 

Most preclinical studies identified a strong link between cellular senescence and mitochondrial dysfunction as the cause of metabolic dysbalance in early stages of NAFLD. In NAFLD mouse models with different dietary regimens, elimination of senescent cell either genetically (INK-ATTAC mouse) or pharmaceutically, by dasatinib plus quercetin, reversed liver fat accumulation, while hepatocyte-specific induction of senescence (alb-Xpg mouse) accelerated steatosis development. The latter mouse model was characterized by mitochondrial dysfunction and reduced mitochondrial fatty acid oxidation capacity [51]. Aged mice, which are prone to accumulate fat in hepatocytes, overexpressed markers for mitochondrial dysfunction such as mitochondrial β-oxidation-related genes including PPARα [52]. In other preclinical studies of NAFLD rodent models, a high-fat diet impaired mitochondrial mass and function [53]. Furthermore, free fatty acids can also induce cellular senescence and lipid accumulation directly by signaling through miR-34a-CDK6 [54]. Autophagy in general and selective autophagy of dysfunctional mitochondria (mitophagy) in particular are considered as protective mechanisms in NAFLD and restoring hepatocytes’ capacity for mitophagy prevents the onset of NAFLD in preclinical models [55,56].

Besides being main energy-producing organelles, mitochondria are also a central source of reactive oxygen species (ROS) [57]. Oxidation of fatty acids by enzymatic break down is a major energy-generating process. If fatty acid provision exceeds the normal (physiological) amount (e.g., by alimentary intake), the resulting enhanced electron flux in mitochondria leads to increased production of ROS and toxic aldehydes causing mitochondrial damage [58]. The link between mitochondrial dysfunction and cellular senescence is known from other chronic diseases [59]. The most important triggers of senescence are ROS, altered Ca^2+^ metabolisms, decreased NAD+, higher AMPK activity, and bioenergetics imbalance [60]. 

In humans and mice with NAFLD, a reduced cellular availability of NAD+ resulted in higher fat content and alimentary NAD+ supplementation reversed the effect [61,62]. Low NAD+ levels may activate p53, thereby inducing cell cycle arrest [63]. ACMSD (α-amino–β-carboxymuconate-ε-semialdehyde) degrades NAD+ and its pharmacological inhibition protected against lipid accumulation and normalized mitochondrial function and ROS production [64]. Calcium ions represent an essential second messenger in multiple cellular processes and fat accumulation in hepatocytes causes changes in the Ca^2+^ homeostasis. This culminates into a vicious cycle of accelerated fat accumulation, cell death and HCC development through ER stress, ROS activation and NAFLD progression [65]. Accumulation of Ca^2+^ e.g., by mutation of ITPR2 (Inositol 1,4,5 Triphosphate receptor type 2) is associated with less senescence and reduced fat in the liver [66]. 

Further molecules and pathways such as mitochondria–nucleus retrograde communication pathway (UPR^mt^) [67], as well as mitochondrial proteases and chaperons such as heat shock protein 60 [68], ClpP (Caseinolytic mitochondrial matrix peptidase proteolytic subunit) [69] and others, have shown an association between mitochondrial dysfunction, senescence and chronic liver disease. However, more work needs to be done to clarify their exact role in NAFLD and also to understand how to target them pharmaceutically. 

#### 2.3.2. The Putative Role of Senescence in the Process of Immune Cell Recruitment 

Disease progression in NAFLD is characterized by inflammation marking the transition to NASH and more severe stages of NAFLD [70,71]. Generally, this process requires the recruitment of monocytes and macrophages [72,73,74]. In depth analyses on how inflammation is triggered in NASH has identified hepatocyte senescence to be a potentially central mediator. After a methionine- and choline-deficient (MCD) diet, wild-type mice expressed high ALT levels and increased apoptotic liver cell death associated with high p53 expression. Reverting the diet to normal chow reduced the local inflammatory response whilst p53 expression remained high, pointing towards senescence as a potential trigger of inflammation during progression from NAFL to NASH [75]. 

HNF4α is a hepatocyte-specific transcription factor regulating multiple cell processes. In mouse models of high-fat diet (HFD) and high-fat high-cholesterol (HFCF) diet, increasing HNF4α expression protected against NASH by reducing expression of genes involved in inflammation, fibrogenesis and senescence (e.g., TNFα, IL6, IL1b, MCP1, F4/80 and p53), liver injury and ROS production. Deletion of p53 in hepatocytes with HNF4α overexpression (AAV8-ALB-hHNF4α) diminished the protective effect, suggesting that HNF4α exerted its effect through p53 [76]. Another study explored the potential global role of p53 in the complex network of pathways and cell–cell interactions involved in mediating injury and local inflammation in chronic liver disease and NAFLD [77]. Global p53 knockout mice exhibited a reduced αSMA, collagen and TGF-β expression and ROS accumulation in the liver upon MCD diet in comparison to WT controls. Authors proposed an increased TGF-β1 signaling, which is, among CCL2, IL6 and others, a central SASP component, as the cause of p53 activation in NAFLD/NASH [77]. The effect of TGF-β1 might be more diverse as other studies showed that this cytokine not only spreads senescence [26] but also activates alternative-type macrophages [78] and hepatic stellate cells [79] in a paracrine manner along with other components of the SASP. In this context, CCR2 is primarily expressed on monocytes, and the corresponding ligand CCL2 is upregulated in NASH. Activation of the CCL2–CCR2 axis and thus recruitment of monocytes and macrophages was observed to be causal for progression of NASH to fibrosis [80]. 

Some other pre-clinical studies elaborated on distinct senescence-associated molecules being involved in metabolic liver diseases. DNA damage checkpoint protein ATM (ataxia-telangiectasia mutated) was activated as a result of reactive oxygen species. Subsequent DNA damage led to apoptotic cell death in a mouse model of fatty liver disease. Its deletion resulted in improvement of apoptosis and fibrosis [81]. Senescence marker protein 30 (SMP30) is an antioxidant protein protecting against apoptosis. Its genetic deletion increased fat accumulation and inflammation in mice upon standard diet [82,83]. 

#### 2.3.3. The Role of Cellular Senescence in Modulating Fibrosis in NASH

Progression of NAFLD is not only driven by inflammation but also characterized by extracellular matrix accumulation and fibrosis. Stellate cell activation by oxidative stress, cytokines and release of damage-associated molecular patterns (DAMPs) from cell death may be central not only for NASH [70,71] but also for other types of chronic liver disease [72,73,74]. Cellular senescence and its role in the interplay between hepatocytes or stellate cells in NAFLD remain insufficiently understood.

Generally, inflammasome activation by DAMPs and PAMPs activates hepatic stellate cells to produce extracellular matrix and leads to an enhanced ECM secretion [84]. Whilst hepatocyte senescence induces SASP and may activate stellate cells through ROS production and secretion of cytokines such as TGF-β1 [85], the spread of cellular senescence from hepatocytes to stellate cells may also lead to inactivation and thus prevention of fibrogenesis [86]. 

Mitochondria-derived DAMPs (mito-DAMPs) released by hepatocytes after mitochondrial injury trigger maturation of stellate cells and circulating levels of mito-DAMPs are significantly increased in NASH [87]. Hepatocyte pyroptosis leading to uncontrolled DAMP release prompts stellate cells to engulf extracellular NLRP3 inflammasome particles. Subsequent cellular activation stimulated IL1b secretion and expression of a-smooth muscle actin (sSMA) [88]. NLRP3 inhibition was shown to reduce fibrosis in rodent NAFLD models [89]. Components of senescent hepatocytes and SAPS such as TGF-β1 and ROS can activate stellate cells, thus inducing fibrosis independent of injury. 

In an in vitro study, hepatocytes incubated with fatty acids induced fat accumulation and β-Gal expression indicated cellular senescence. Subsequent co-culture activated stellate cells and increased ECM secretion. This process was regulated by antioxidant regulator nuclear factors erythroid 2-related factor 2 (Nrf2). Its upregulation mitigated oxidative stress, cellular senescence and stellate cell activation [88]. 

However, cellular senescence may also spread over the tissue among different cell types. Senescent stellate cells are silenced and are considered beneficial. Insulin-like growth factor 1 (IGF-1) is among a multitude of growth factors, which exert pleiotropic effects in liver disease. Generally, IGF-1 can regenerate liver injury. In a mouse model of methionine-choline-deficient diet, IGF-1 reduced fibrosis by making stellate cells senescent and it restored mitochondrial dysfunction and reduced oxidative stress in hepatocytes. The protective effect of IGF-1 in stellate cells was minimized in a global p53 knockout mouse [89], principally proving that IGF-1 exerts its effects via p53-driven senescence (Table 2). 

## 3. Summary and Outlook

In summary, there are substantial data suggesting that senescence plays an important role in NAFLD development and disease progression. Human data underline that markers of senescence such as telomere length, DNA damage, DNA methylation and activation of p53-p21 pathway are linked with fat accumulation, inflammation and fibrogenesis. 

**Figure 3:**
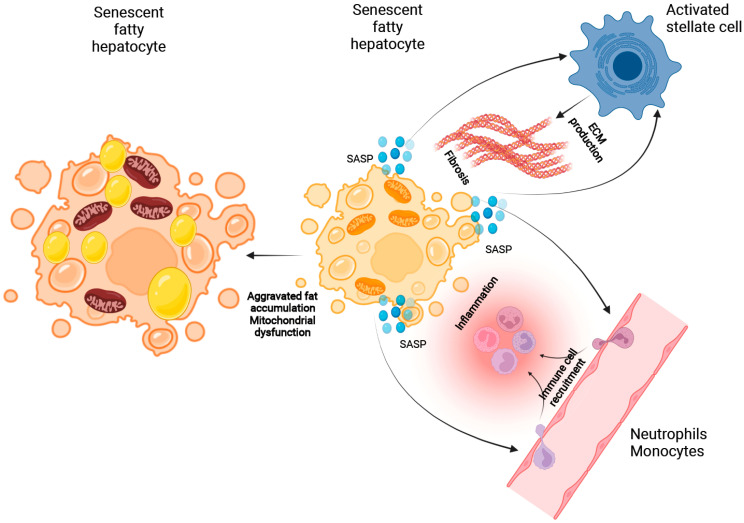
The potential role of cellular senescence in NAFLD progression. Hepatocellular senescence and mitochondrial dysfunction may aggravate fat accumulation in hepatocytes. The senescence-associated secretory phenotype (SASP) initiates recruitment of monocytes and neutrophils, thereby inducing inflammation and transition to NASH. In the same manner, SASP activates hepatic stellate cells leading to fibrogenesis.

Our (detailed) mechanistic understanding on how senescence is involved in disease mechanisms is currently incomplete. During the first phase of fat accumulation, the interplay between cellular senescence and mitochondrial dysfunction may aggravate disease patterns, increasing the fat content. ROS as well as a disturbed NAD+ and Ca^2+^ homoeostasis trigger mitochondrial dysfunction. Thus, NAFL and p53-dependent hepatocyte senescence after high-calorie diet establishes SASP to recruit immune cells that promote NASH development. Involvement of cellular senescence in processes related to fibrogenesis and stellate activation/deactivation is less clear, but stellate cell stimulation as the consequence of hepatocyte SASP may act as a pro-fibrotic mechanism (Figure 3). 

Although these studies described the potential role of cellular senescence in NAFLD, there is a persistent lack of understanding of how hepatocellular senescence is precisely linked to NAFLD progression, and important questions remain unanswered. We are still searching for the molecular triggers balancing senescence vs. alternative cell responses (e.g., cell death) as well as for the exact sequence of how disease phenotypes such as intracellular lipid accumulation, hepatocellular senescence and immune cell recruitment develop during disease progression. This will be essential in order to target senescence therapeutically at different disease stages. 

It is therefore tremendously important to explore senescence-related pathomechnisms in NAFLD and NASH in order to identify novel therapeutic options, which may halt disease progression and revert pathological processes. 

## Figures and Tables

**Table 1:** Human studies exploring the phenotype of senescence in fatty liver disease.

Author	Specimens	Experimental Techniques	Results
Panasiuk A et al. [37]	Liver (n = 84)	IHC ^1^	Liver steatosis is associated with p53 expression and increasing pro-apoptotic BAX/BCL-2 ratio
Ping F et al. [38]	PBMC (blood) (n = 70)	rtPCR ^2^	Telomere length in leukocytes shortened in patients with diabetes type 2 who developed NAFLD
Laish I et al. [40]	PBMC (blood) (NAFLD n = 22; crypt. Cirrhosis n = 20; healthy n = 20)	FISH ^3^, rtPCR ^2^	Shorter telomere length and decreased expression of telomerase reverse transcriptase in NAFLD
Aravinthan A et al. [41]	Liver (NAFLD n = 70; healthy n = 60)	FISH ^3^, IHC ^1^	NAFLD and degree of fibrosis was associated with shorter telomeres, cellular senescence (p21) and DNA damage (yH2AX)
Akazawa Y et al. [42]	Liver (NAFLD n = 43; healthy n = 9)	IF ^4^	The number of foci with DNA double strand breaks (53BP1) increases with NAFLD and progression to NASH
Pirola CJ et al. [43]	Liver (NAFLD n = 252)	MT-CYB ^5^ sequencing; differential mtDNA ^6^ damage; global liver transcriptome, profiling circulating Krebs cycle metabolites; tissue levels products of lipid peroxidation and markers of oxidative stress	NASH was associated with higher MT-CYB ^5^ variance and changes in global liver transcriptome; liver mtDNA ^6^ damage, tissue levels of oxidative adducts and lipid peroxyl radicals were associated with advanced fibrosis
Ahrens M et al. [44]	Liver (NAFLD n = 45; healthy n = 18)	Array based DNA methylation and mRNA ^7^ expression	NAFLD associated with methylation differences in nine genes coding for enzymes in metabolism and insulin signaling. Methylation signatures partially reversible after bariatric surgery
Hotta K et al. [45]	Liver (NAFLD n = 60)	Genome-wide DNA methylation levels measured by the Illumina Infinium HumanMethylation450 BeadChip	Two differentially methylated region networks involved in NAFLD progression: 1. Genes involved in transcriptional regulation, cytoskeleton, proliferation; 2. Genes associated with metabolic pathways
Johnson ND et al. [46]	Liver (NAFLD n = 325)	Infinium MethylationEPIC array	DNA methylation associated with fibrosis progression with increasing proportion of natural killer cells
Hardy T et al. [47]	Liver and Plasma (NAFLD n = 26)	Plasma cell-free DNA methylation of PPARy ^8^—pyrosequencing Liver DNA methylation—laser capture microdissection and pyrosequencing	Differential DNA methylation at PPARy ^8^ promotor detectable in circulating cell free DNA as a non-invasive marker
Aravinthan A et al. [48]	PBMCs from two cohorts of NAFLD (n = 323, n = 123)	p21 polymorphisms (SNP ^9^)—genotyping	SNP rs762623 significantly associated with disease progression

^1^ IHC—immunohistochemistry. ^2^ rtPCR—real-time polymerase chain reaction. ^3^ FISH—fluorescence in-situ hybridization. ^4^ IF—immunofluorescence. ^5^ MT-CYB—mitochondrially encoded cytochrome B. ^6^ mtDNA—mitochondrial deoxyribonucleic acid. ^7^ mRNA—messenger ribonucleic acid. ^8^ PPARy—peroxisome proliferator-activated receptor gamma. ^9^ SNP—single nucleotide polymorphism.

**Table 2:** Preclinical in vivo studies exploring mechanisms of senescence in fatty liver disease.

Author	Species	Model	Results
Zhang G et al. [50]	Rats (Crl:CD (SD) rats)	HF diet ^1^	Increasing activation of p21 and p16 pathways in livers with fat accumulation
Ogrodnik M et al. [51]	Mice (C57BL/6; INK-ATTAC.; Alb-Xpg)	Aged mice fed ad libitum	Cellular senescence drives hepatic steatosis and senolysis reverts the effect
Wan J et al. [52]	Mice (C57BL/6)	Aged mice fed with HF diet ^1^ or normal chow	Increasing liver fat accumulation with higher age related to upregulation of RAGE and inhibition of PPARα
Lohr K et al. [53]	Mice (C57BL/6)	Aged mice fed with HF diet ^1^	Reduced mitochondrial mass and function foster fatty liver development
Qin YE et al. [54]	Mice (C57BL/6)	HF diet ^1^	Liver steatosis induced hepatocyte senescence through miR-34a by targeting CDK6
Han X et al. [61]	Mice (C57BL/6)	Aged mice fed with normal chow	NAD precursor nicotinamid riboside (NR) has protected from aging-induced NAFLD
Li DJ et al. [62]	Mice (C57BL/6; Fndc5^−/−^)	HF diet ^1^ and MCD diet ^2^	Nicotinamid riboside (NR) exerts its protective effect through Fndc5/irisin upregulation
Archer AE et al. [68]	Wistar rats	HF diet ^1^ and heat treatment (41 °C)	Heat shock protein 72 improves glucose tolerance and reduces triglyceride storage
Bhaskaran S et al. [69]	Mice (C57BL/6; ClpP^−/−^)	HF diet ^1^	Caseinolytic peptodase P (ClpP) regulated mitochondrial function and its deficiency protects from fat accumulation in the liver
Farrell GC et al. [75]	Mice (C57BL/6)	MCD diet ^2^	MCD liver fat accumulation promotes p53 expression and subsequent apoptosis
Xu Y et al. [76]	Mice (C57BL/6; hepatocyte specific p53^−/−^)	HFCF diet ^3^	HNF4a prevents hepatic triglyceride accumulation and promotes fatty acid oxidation but not in hepatocyte-specific p53^−/−^ mice
Tomita K et al. [77]	Mice (C57BL/6; p53^−/−^)	MCD diet ^2^	p53 promotes lipid peroxidation, apoptotic hepatocytes and progression of NADLF in a TGF-b-dependent manner
Daugherity EK et al. [81]	Mice (C57BL/6; Atm^−/−^)	HF diet ^1^	Dietary liver fat induced ROS production and DNA damage which leads to apoptosis and fibrosis in a ATM dependent manner
Kondo Y et al. [82]	Mice (Lepr^db/db^Smp30^Y/−^)	Normal chow (aged mice)	Senescence marker protein-30 (SMP30) increases oxidative stress and liver inflammation together with PPARa induction and fat accumulation
Kondo Y et al. [83]	Mice (SMP30/SOD1-DKO)	Normal chow	Dual deficiency of SMP30 and SOD1 promotes liver fat accumulation and inflammation
Mridha AR et al. [89]	Mice (foz/foz; C57BL/6)	MCD diet ^2^	NLRP3 inflammasome stimulation promotes inflammation and fibrosis in NAFLD
Nishizawa H et al. [90]	Mice (C57BL/6; db/db)	MCD diet ^2^	IGF-1 prevents from fibrosis in NASH by inducing cellular senescence to HSC through p53

^1^ HF diet—high-fat diet. ^2^ MCD diet—methionine- and choline-deficient diet. ^3^ HFCH diet—high-fat/cholesterol/fructose diet.

## Data Availability

Not applicable.
